# fMRI functional connectivity of the periaqueductal gray in PTSD and its dissociative subtype

**DOI:** 10.1002/brb3.579

**Published:** 2016-09-20

**Authors:** Sherain Harricharan, Daniela Rabellino, Paul A. Frewen, Maria Densmore, Jean Théberge, Margaret C. McKinnon, Allan N. Schore, Ruth A. Lanius

**Affiliations:** ^1^ Department of NeuroscienceUniversity of Western OntarioLondonONCanada; ^2^ Department of PsychiatryUniversity of Western OntarioLondonONCanada; ^3^ Department of PsychologyUniversity of Western OntarioLondonONCanada; ^4^ Imaging DivisionLawson Health Research InstituteLondonONCanada; ^5^ Departments of Medical Imaging and Medical BiophysicsWestern UniversityLondonONCanada; ^6^ Mood Disorders ProgramSt. Joseph's HealthcareHamiltonONCanada; ^7^ Department of Psychiatry and Behavioural NeurosciencesMcMaster UniversityHamiltonONCanada; ^8^ Homewood Research InstituteGuelphONCanada; ^9^ Department of Psychiatry and Biobehavioral SciencesUniversity of California at Los AngelesLos AngelesCAUSA

**Keywords:** active defenses, autonomic blunting, cingulate gyrus, fight–flight, fusiform gyrus, hyperarousal, insula, passive defenses, periaqueductal gray, posttraumatic, temporoparietal junction

## Abstract

**Background:**

Posttraumatic stress disorder (PTSD) is associated with hyperarousal and active fight or flight defensive responses. By contrast, the dissociative subtype of PTSD, characterized by depersonalization and derealization symptoms, is frequently accompanied by additional passive or submissive defensive responses associated with autonomic blunting. Here, the periaqueductal gray (PAG) plays a central role in defensive responses, where the dorsolateral (DL‐PAG) and ventrolateral PAG (VL‐PAG) are thought to mediate active and passive defensive responses, respectively.

**Methods:**

We examined PAG subregion (dorsolateral and ventrolateral) resting‐state functional connectivity in three groups: PTSD patients without the dissociative subtype (*n *= 60); PTSD patients with the dissociative subtype (*n *= 37); and healthy controls (*n *= 40) using a seed‐based approach via PickAtlas and SPM12.

**Results:**

All PTSD patients showed extensive DL‐ and VL‐PAG functional connectivity at rest with areas associated with emotional reactivity and defensive action as compared to controls (*n *= 40). Although all PTSD patients demonstrated DL‐PAG functional connectivity with areas associated with initiation of active coping strategies and hyperarousal (e.g., dorsal anterior cingulate; anterior insula), only dissociative PTSD patients exhibited greater VL‐PAG functional connectivity with brain regions linked to passive coping strategies and increased levels of depersonalization (e.g., temporoparietal junction; rolandic operculum).

**Conclusions:**

These findings suggest greater defensive posturing in PTSD patients even at rest and demonstrate that those with the dissociative subtype show unique patterns of PAG functional connectivity when compared to those without the subtype. Taken together, these findings represent an important first step toward identifying neural and behavioral targets for therapeutic interventions that address defensive strategies in trauma‐related disorders.

## 1 Introduction

Posttraumatic stress disorder (PTSD) involves reexperiencing, avoidance, and hyperarousal symptoms, where individuals tend to be hypervigilant of their surroundings to ensure their own safety and to avoid exposure to threatening stimuli (American Psychiatric Association, 2013; Dalgleish, Moradi, Taghavi, Neshat‐Doost, & Yule, 2001; Ehlers & Clark, 2000; Taylor, Kuch, Koch, Crockett, & Passey, 1998; van der Kolk & McFarlane, 1998, Yehuda et al., 2015). When a threat is detected, PTSD patients may display hyperarousal symptoms associated with active defensive fight or flight circuitry of the sympathetic nervous system as evidenced by increased heart rate, skin conductance, and blood pressure (Pole, 2007). By contrast, patients with the less common dissociative subtype of PTSD (14%) (Stein et al., 2013), characterized by symptoms of depersonalization and derealization, often exhibit passive or submissive defensive responses accompanied by autonomic blunting (Corrigan, Fisher, & Nutt, 2011; Lanius, Bluhm, Lanius, & Pain, 2006; Lanius et al., 2005, 2010).

Schauer and Elbert (2010) recently proposed a defense cascade model aimed at explaining the typical defensive reaction of an organism. Here, the presence of dissociative states in humans exposed to trauma is associated with a transition from fight or flight defensive responses to more primitive animal defensive responses. These defensive responses, evoked in passive or submissive responses to threats, include unresponsive immobility, emotional blunting, and analgesia (Baldwin, 2013; Nijenhuis, Vanderlinden, & Spinhoven, 1998; Porges, 1995). Interestingly, Bandler, Keay, Floyd, and Price (2000) propose that the periaqueductal gray (PAG), a small structure in the midbrain that consists of multiple subdivisions that oppose each other in function, is a central structure for mediating autonomic responses and is thus responsible for coordinating defensive reactions when confronted with threatening stimuli. Specifically, Bandler et al. suggest that whereas the dorsolateral and lateral periaqueductal gray (DL‐PAG and L‐PAG) are associated with sympathetic nervous system activation that evokes active defensive strategies, the ventrolateral PAG (VL‐PAG) is associated with passive coping strategies via activation of the parasympathetic nervous system. A recent preclinical study by Adamec, Toth, Haller, Halasz, and Blundell (2012) supported this hypothesis, where the dorsolateral PAG was associated with anxiety‐related responses to stress in rodents. By contrast, the ventrolateral PAG exhibited a contrasting immobility or passive reaction to stress.

Kozlowska, Walker, McLean, and Carrive (2015) explicitly applied the functions of the PAG subdivisions to the defense cascade model (Schauer & Elbert) suggesting that when a threat is detected, DL‐PAG and L‐PAG subdivisions coordinate hyperarousal symptoms, such as fight or flight responses associated with sympathetic nervous system activity in response to threat. Here, it is believed that endocannabinoids facilitate further release of cortisol to elicit an acute stress response from the organism. Concomitant activation of the locus coeruleus in the brainstem may induce vasoconstriction of peripheral blood vessels and thus increase blood supply to muscles that would allow the organism to fight the predator (George et al., 2013; Goadsby, Lambert, & Lance, 1985; Gorzalka, Hill, & Hillard, 2008; Patel, Roelke, Rademacher, Cullinan, & Hillard, 2004). In cases where the threat becomes inescapeable, the VL‐PAG predominates as parasympathetic nervous system activation overrides sympathetic nervous system activation through increased vagal efferents from the dorsal motor nucleus, which in turn may produce hypoarousal symptoms that cause a freezing or submissive shutdown response, sometimes referred to as ‘conservation withdrawal’ (An, Bandler, Ongur, & Price, 1998; Porges, 2001). Projections from the VL‐PAG to the medulla may play a role in generating defensive freezing behavior (Tovote et al., 2016), which may be associated with the recruitment of presynaptic opioid receptors that mediate analgesic relief (Musha, Satoh, Koyanagawa, Kimura, & Satoh, 1989).

Previous functional imaging studies of the PAG (Linnman, Moulton, Barmettler, Becerra, & Borsook, 2012) have supported functional segregation of the structure into multiple subdivisions that vary in function, with the dorsal PAG associated with elevated blood pressure and the ventral PAG stimulating lower blood pressure and parasympathetic dominance. In particular, in resting‐state functional connectivity studies of the PAG in healthy populations, connectivity has been observed with the cerebellum subcortical network as well as the thalamus and the amygdala (Tomasi & Volkow, 2011). Critically, however, the complex neural circuitry of the PAG has not yet been delineated in PTSD and its dissociative subtype.

Accordingly, the aim of this study was to examine resting‐state functional connectivity patterns of the PAG subdivisions in PTSD, as it was hypothesized that this patient population would exhibit greater defensive posturing even during the resting state. An additional aim was to compare patterns of activation between individuals with and without the dissociative subtype of PTSD. We hypothesized that all PTSD patients would demonstrate increased functional connectivity of both PAG subdivisions with brain regions involved in threat appraisal (dorsal anterior cingulate cortex, fusiform gyrus) (see Milad et al., 2007; Porges, 2007). Moreover, given that both PTSD and its dissociative subtype are associated with fight–flight and concomitant hyperarousal responses, we hypothesized that both groups would demonstrate increased DL‐PAG functional connectivity with brain structures associated with sympathetic nervous system activity and consequent active defensive strategies, including the anterior insula and premotor cortex (see Butler et al., 2007; Critchley, Nagai, Gray, & Mathias, 2011). We hypothesized, however, that only those with the dissociative subtype of PTSD would demonstrate VL‐PAG functional connectivity with brain structures associated with depersonalization and passive defensive responses, including the temporoparietal junction and the rolandic operculum (see Blanke & Arzy, 2005; Daniels, Frewen, Théberge, & Lanius, 2016; Zaytseva et al., 2015).

## 2 Methods

### 2.1 Participants

One‐hundred and thirty‐seven age‐matched subjects were included in the study: 60 patients with a primary diagnosis of PTSD without the dissociative subtype (PTSD‐DS), 37 PTSD patients with the dissociative subtype of PTSD (PTSD + DS), and 40 healthy controls. The participants were recruited by the LHSC (London Health Sciences Centre) Department of Psychiatry during 2009–2016 via referrals from family physicians, mental health professionals, psychology/psychiatry clinics, community programs for traumatic stress survivors, and posters/advertisements within the London, Ontario community.

A primary PTSD diagnosis was determined using the CAPS‐IV (Clinician‐Administered PTSD Scale), which assesses 17 categorized symptoms associated with PTSD on separate frequency and intensity scales, with the diagnosis confirmed by the DSM‐IV criteria with an additional minimum severity score of 50 (Blake et al., 1995). PTSD patients with the dissociative subtype had the additional requirement of scoring at least two on both the frequency and intensity scales for depersonalization or derealization symptoms (as per Nicholson et al., 2015 and Steuwe et al., 2014). For each participant, comorbid Axis‐I disorders were diagnosed with the SCID (Structure Clinical Interview for DSM‐IV Axis‐I disorders) (First, Spitzer, Gibbon, & Williams, 2002). A battery of questionnaires was also administered, including the Beck Depression Inventory (BDI; Beck, Guth, Steer, & Ball, 1997) to assess depression symptoms, the Child Trauma Questionnaire (CTQ; Bernstein et al., 2003) to assess childhood trauma history [92 PTSD patients (85% of PTSD‐DS and PTSD+DS) who met criteria for interpersonal childhood trauma according to CTQ cut‐off scores] (Bernstein & Fink, 1998; DiLillo et al., 2006), and the Multiscale Dissociative Inventory (MDI; Briere, Weathers, & Runtz, 2005) to assess further dissociative experiences.

Exclusion criteria for all participants included metal implants that violate 3.0T scanner safety regulations, a previous head injury associated with loss of consciousness, current or past history of neurological disorders, significant untreated medical illness, and pervasive developmental mental disorders. PTSD patients were excluded if they met criteria for current or past history of bipolar or psychotic disorders, or if patients had alcohol/substance dependency or abuse that had not sustained full remission for at least 6 months prior to study entry. Control participants were excluded if lifetime criteria were met for any DSM‐IV Axis‐I psychiatric disorder.

All scanning was conducted at either Robarts Research Institute's Center for Functional and Metabolic Mapping or Lawson Health Research Institute in London, Ontario, Canada. The study was approved by the Research Ethics Board at Western University of Canada. All participants provided written informed consent to partake in the study.

### 2.2 Data acquisition

Whole‐brain fMRI (functional magnetic resonance) data were obtained using a 3.0T scanner (Magnetom Tim Trio, Siemens Medical Solutions, Erlangen, Germany) with a 32‐channel phased array head coil where the participant's head was supported with foam padding. BOLD (blood oxygen level dependent) fMRI data were collected using a manufacturer's standard gradient‐echo planar imaging (EPI) pulse sequence (single‐shot, blipped‐EPI) with an interleaved slice acquisition order with the following parameters: Time Resolution (TR)  = 3000 ms, Echo Time (TE) = 20 ms, voxel size = 2 × 2 × 2 mm^3^, Field of View (FOV)  = 192 × 192 × 128 mm^3^ (94 × 94 matrix, 64 contiguous slices), and Flip Angle (FA)  = 90°. High‐resolution T1‐weighted anatomical images were also obtained (MPRage: 192 slices, voxel size = 1 × 1 × 1 mm^3^). To obtain resting‐state data, subjects were asked to close their eyes and let their minds wander without focusing on anything in particular for 6 min (as per standard methods in Fransson, 2005; also see Bluhm et al., 2009).

### 2.3 Resting‐state fMRI data preprocessing

Image preprocessing and statistical analyses were performed using statistical parametric mapping software (SPM12, Wellcome Trust Center for Neuroimaging, London, UK: www.fil.ion.ucl.ac.uk/spm; RRID:SCR_007037) within MATLAB 8.6 (R2015b, Mathworks Inc., MA; RRID:SCR_001622). Four dummy scans were omitted from the fMRI time series to allow magnetization reach steady state before the experiments commenced and enhance the quality of realignment during image preprocessing. The functional images for each subject were realigned to the first functional image to correct for motion in the scanner and resliced. The mean functional image was created and subsequently coregistered to the T1‐weighted structural image for each subject to spatially realign functional images to the subject's anatomical space. The coregistered images were segmented into gray matter, white matter, cerebrum spinal fluid, bone, soft tissue, and air using the “New Segment” method implemented in SPM12, which uses T2‐weighted and PD‐weighted scans when generating tissue probability maps. The resulting forward deformation fields were generated and used to spatially normalize the functional images to MNI space without resampling the voxel size, and each subject was visually inspected to ensure precise normalization patterns given the small anatomical region being studied. The images were then smoothed with a three‐dimensional isotropic Gaussian kernel of 4 mm FWHM (full‐width at half‐maximum), in coordinance with a previous PAG functional neuroimaging study (Dunckley et al., 2005) and a PAG neuroimaging meta‐analysis (Linnman et al., 2012) that suggested using a lower smoothing kernel facilitates higher voxel resolution and thus helps elicit optimal functional connectivity patterns based on a smaller neuroanatomical area in the brain (Becerra, Harter, Gonzalez, & Borsook, 2006). Beissner, Deichmann, and Baudrexel (2011) investigated optimal smoothing and normalization patterns in the brainstem and also found that a relatively lower smoothing kernel may be necessary to obtain significant results given its small region in the brain. It is important to note that this study still satisfies the theory of Gaussian fields developed by Friston et al. (1995), which recommends that Gaussian smoothing should be a least double the voxel size (2 mm voxel size to 4 mm smoothing).

The smoothed functional images were further motion corrected with ART software (version 2015‐10; Gabrieli Lab, McGovern Institute for Brain Research, Cambridge, MA; http://www.nitrc.org/projects/artifact_detect/; RRID:SCR_005994) at a motion threshold of 2 mm, as motion artifacts may significantly affect the BOLD signal in resting‐state functional connectivity studies (Power, Barnes, Snyder Schlaggar, & Petersen, 2012). The outlier motion regressors identified with ART were used as a covariate of no interest during within‐subject (first‐level) analysis. The smoothed functional images were subsequently bandpass filtered to reduce the signal‐to‐noise ratio using 0.012 and 0.1 Hz as the high‐pass and low‐pass frequency cut‐offs, respectively (in‐house software by coauthor Jean Théberge, Lawson Health Research Institute).

### 2.4 Seed‐based regions of interest

**Figure 1:**
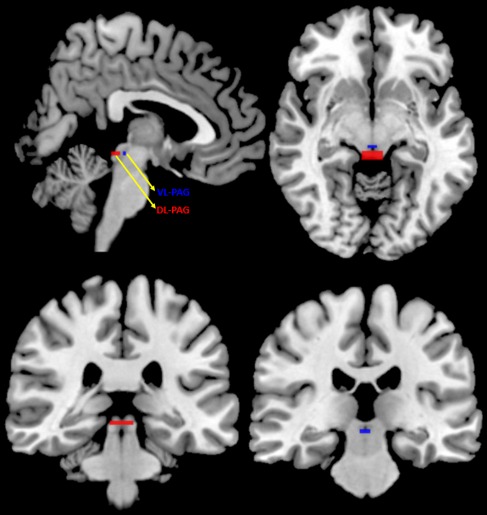
Dorsolateral and Ventrolateral PAG Regions of Interest. Two box‐shaped masks were created to define both the dorsolateral (DL‐PAG, red; MNI x: 0; y: −32; z: −8.5 plus 6 × 2  × 1.5 mm extensions) and ventrolateral (VL‐PAG, blue; MNI x: 0; y: −27; z: −8 plus 3 × 1 × 1 mm extensions) subdivisions of the PAG. These masks are presented in sagittal (top left), axial (top right), and coronal (bottom) views

Seed region‐of‐interest masks (ROI) were generated using PickAtlas software (WFU Pickatlas, version 3.0.5; Maldjian, Laurienti, Kraft, & Burdette, 2003; http://fmri.wfubmc.edu/software/pickatlas; RRID:SCR_007378) in coordinance with an atlas developed by Ezra, Faull, Jbabdi, and Pattinson (2015), which mapped the PAG subdivisions via a column tractography study using diffusion MRI. Two box‐shaped masks were created to define both the dorsolateral (MNI x: 0; y: −32; z: −8.5 plus 6 × 2 × 1.5 mm extensions) and ventrolateral (MNI x: 0; y: −27; z: −8 plus 3 × 1 × 1 mm extensions) subdivisions of the PAG (Fig. 1).

### 2.5 Statistical analyses

#### 2.5.1 Demographics and psychological measures

The demographic and clinical characteristics of study participants are outlined in Table 1. A one‐way ANOVA was performed to assess age differences across participant groups, and a Pearson's chi‐square test was used to determine the effect of gender differences across all three participant groups. A Kruskal–Wallis analysis assessed the normal distribution of nonparametric psychological measures (CAPS, BDI, CTQ, and averaged depersonalization and derealization scores from MDI) with post hoc tests to determine significant differences between groups (Kruskal & Wallis, 1952).

**Table 1:** Demographic and clinical information. Age, gender, CAPS, and self‐report questionnaires (CTQ, MDI, BDI) are reported as mean ± *SD*. Psychiatric disorders assessed via SCID‐I (MDD, Panic Disorder/Agoraphobia, Social Phobia, OCD and GAD) are reported in frequencies, as *n *= current (past) cases

Measure	PTSD‐DS	PTSD + DS	Controls
N	60	37	40
Age	37.8 ± 11.6	40.4 ± 13.7	35.0 ± 11.0
Gender	M = 25, F = 35	M = 8, F = 29	M = 14, F = 26
CAPS—Total	67.9 ± 13.4	81.6 ± 12.7	0.7 ± 3.1
CTQ—Total	56.3 ± 24.7	68.2 ± 19.1	31.6 ± 8.6
BDI	22.8 ± 7.5	33.0 ± 10.3	1.2 ± 2.1
MDI—Total	54.1 ± 15.2	77.2 ± 22.0	33.7 ± 3.4
MDI–Depersonalization	6.6 ± 2.7	12.0 ± 5.2	5.2 ± 0.6
MDI–Derealization	8.6 ± 3.4	12.7 ± 4.0	5.2 ± 0.5
MDD	*n *= 11 (24)	*n *= 23 (9)	–
Panic Disorder/Agoraphobia	*n *= 10 (6)	*n *= 9 (6)	–
Social Phobia	*n *= 2 (2)	*n *= 6 (0)	–
OCD	*n *= 3 (2)	*n *= 0 (2)	–
GAD	*n *= 1 (0)	*n *= 0 (0)	–

PTSD‐DS, nondissociative posttraumatic stress disorder patients; PTSD + DS, dissociative posttraumatic stress disorder patients; M, Males; F, Females; CAPS, Clinician‐Administered PTSD Scale; CTQ, Child Trauma Questionnaire; BDI, Beck Depression Inventory; MDI, Multiscale Dissociation Inventory; MDD, Major Depression Disorder; OCD, Obsessive Compulsive Disorder; GAD, Generalized Anxiety Disorder.

#### 2.5.2 Within‐subject analyses

The masks created in PickAtlas generated tables that provided seed activity of both ROIs for each subject based on whole‐brain resting‐state data. In‐house software developed by coauthor Dr. Jean Théberge read these tables and generated a mean signal intensity time course to be used in a within‐subject multiple regression model along with ART movement regressors. In addition, means of the number of outliers per subject in each group were compared in an effort to examine the potential influence they may have on any findings. Functional connectivity was then assessed using a voxel‐wise approach by calculating both positive and negative correlations between ROIs and other voxels of the brain.

#### 2.5.3 Between‐subject analyses

A whole‐brain 3 (subject group) × 2 (ROI) full‐factorial analysis of variance (ANOVA) was conducted for the between‐subject analyses, with and without using MDD diagnosis as a covariate (MDD was diagnosed via SCID assessment for Axis‐I psychiatric disorders, see 2; Table 1). The between‐group factor consisted of three levels: nondissociative PTSD patients (PTSD‐DS), dissociative PTSD patients (PTSD + DS), and healthy controls, whereas the within‐group factor consisted of two levels: DL‐PAG and VL‐PAG. To determine significant clusters, a family‐wise error (FWE) whole‐brain cluster‐corrected (*p *<* *.05, *k *= 50) threshold was set for both interaction and post hoc analyses. One‐sample *t*‐tests were used to assess connectivity patterns within each group and ROI, whereas two‐sample *t*‐tests assessed between‐group comparisons for both the DL‐PAG and VL‐PAG as well as the differences between both ROIs. Brain regions were identified using the AAL atlas (Tzourio‐Mazoyer et al., 2002) via xjview software ( http://www.nitrc.org/projects/xjview) and visually inspected using another anatomical atlas focusing on a dissected brain (Montemurro & Bruni, 1988). To more accurately distinguish between relevant anatomical areas in close proximity, such as the rolandic operculum and insula, masks of each area were created using PickAtlas software according to the AAL atlas and were inspected to ensure proper identification of brain regions. Brodmann areas of these brain regions were also identified using xjview software and the MNI2Tal atlas available online via the BioImage Suite at Yale University ( http://bioimagesuite.yale.edu/mni2tal/; Lacadie, Fulbright, Constable, & Papademetris, 2008).

#### 2.5.4 Correlations

Correlations between the fMRI data and clinical PTSD symptoms were examined by regressing CAPS (reexperiencing, avoidance. and hyperarousal subscales, in addition to total CAPS score in all PTSD patients), CTQ, and MDI scores. Subsequent ROI analyses were carried out specifically for the left and the right fusiform gyrus (left: MNI x: −46, y: −42, z: −12; right: MNI x: 54, y: −38, z: −16) based on a meta‐analysis of previous neuroimaging studies in PTSD (Patel, Spreng, Shin, & Girard, 2012). Each ROI analysis was conducted independently, drawing a 15‐mm‐radius sphere around the given peak coordinate corrected at FWE *p* <.05 (cluster and peak corrected). Significant correlations were evaluated at FWE *p *<* *.05 (cluster and peak corrected), with subsequent Pearson's correlation coefficients (*r)* calculated between clinical scores and the ROI used in the analysis, as defined above.

## 3 Results

### 3.1 Demographic and clinical measures

ANOVA analyses did not reveal significant differences in ages across all three participant groups (*p *=* *.148, *df* = 2), and a Pearson's chi‐square test revealed no statistically significant association between gender and participant group (*p *=* *.129, *df* = 2). Kruskal–Wallis analysis of variance yielded significant values for all psychological measures, including CTQ, CAPS, MDI, and BDI (all *p *<* *.001). Post hoc Games‐Howell comparisons revealed no significant differences between PTSD + DS and PTSD‐DS groups for CAPS (*p *=* *.794) and BDI scores, but did reveal significantly higher CTQ and MDI (averaged depersonalization and derealization score) scores in PTSD + DS individuals (*p *<* *.05). All psychological measures revealed significantly higher scores in both PTSD patient groups as compared to controls (all *p *<* *.001). In addition, the mean number of outlier functional volumes per subject did not significantly differ across groups (*p *=* *0.327).

### 3.2 Full factorial design

The whole‐brain analysis of variance (3 × 2 ANOVA) revealed an interaction between group and region of interest, with significant main effects observed for each factor (see supplemental results). Using MDD diagnosis as a covariate did not change the results. Post hoc one‐sample and two‐sample *t*‐tests based on the full‐factorial ANOVA were carried out to assess differences observed within and between each variable of the two main factors, group (PTSD + DS, PTSD‐DS, control), and PAG region (DL and VL‐PAG) at *p *<* *.05 (FWE whole‐brain cluster corrected, *k *= 50).

### 3.3 Functional connectivity of DL‐PAG and VL‐PAG within control, nondissociative PTSD, and dissociative PTSD groups

#### 3.3.1 Control subjects

**Figure 2:**
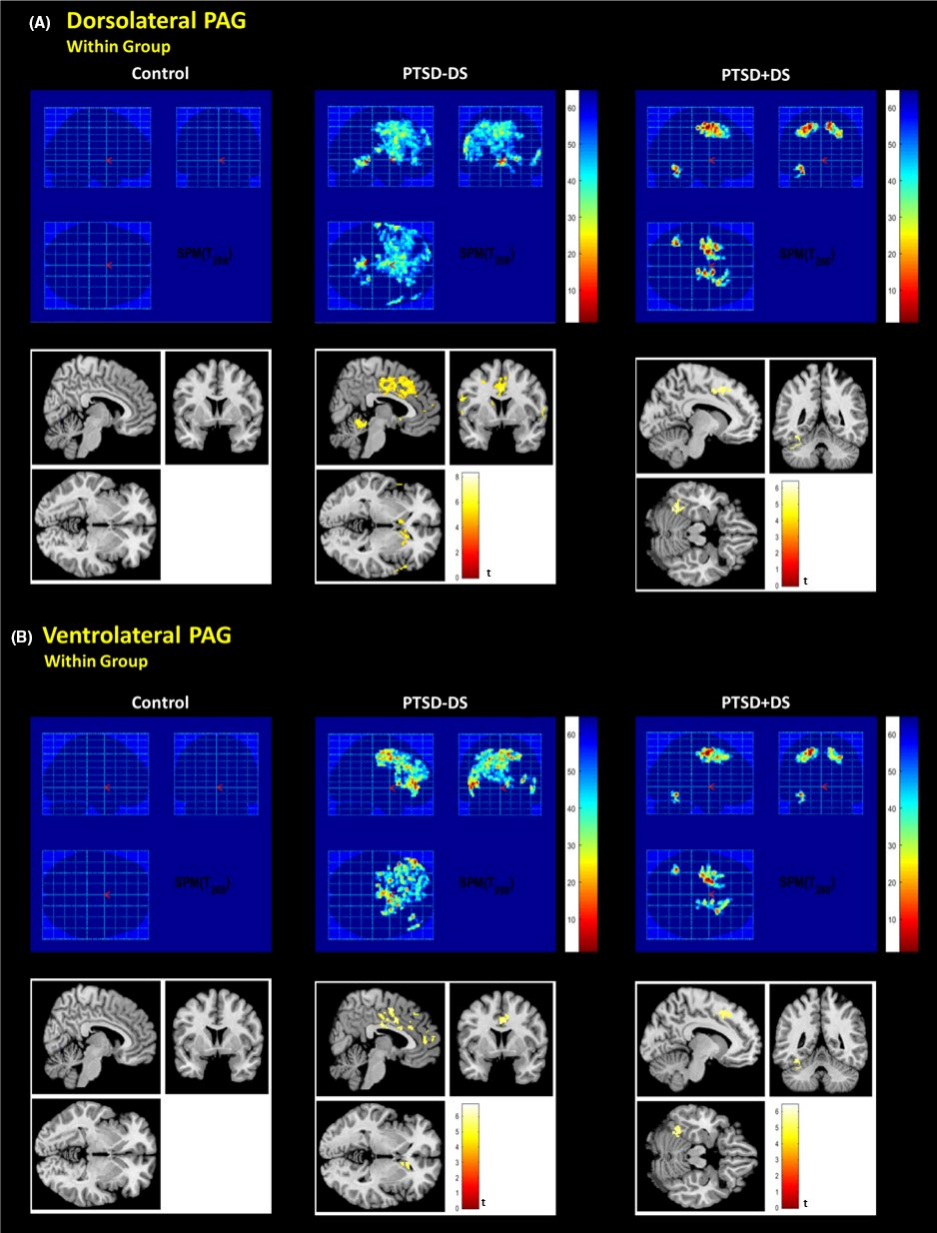
Dorsolateral and ventrolateral PAG connectivity within each subject group during resting state. FWE whole‐brain corrected *p *< .05, *k *= 50; shown at x: 6, y: 0, z: 0, for PTSD‐DS and x: −10, y: −52, z: −16 for PTSD + DS based on MNI coordinates. PTSD‐DS, nondissociative posttraumatic stress disorder patients; PTSD + DS, dissociative posttraumatic stress disorder patients

Control subjects demonstrated DL‐PAG functional connectivity only with the left cerebellar lobule IV. Furthermore, VL‐PAG connectivity was not observed beyond the VL‐PAG itself (Fig. 2; see supplemental results). When comparing the functional connectivity of the DL‐PAG to the VL‐PAG (DL‐PAG > VL‐PAG), there was greater functional connectivity observed with the left cerebellar lobules IV and V, along with the cerebellar vermis. No greater functional connectivity was observed for the VL‐PAG versus the DL‐PAG (FWE whole‐brain cluster corrected at *p *<* *.05, *k *= 50).

#### 3.3.2 PTSD‐DS

**Figure 3:**
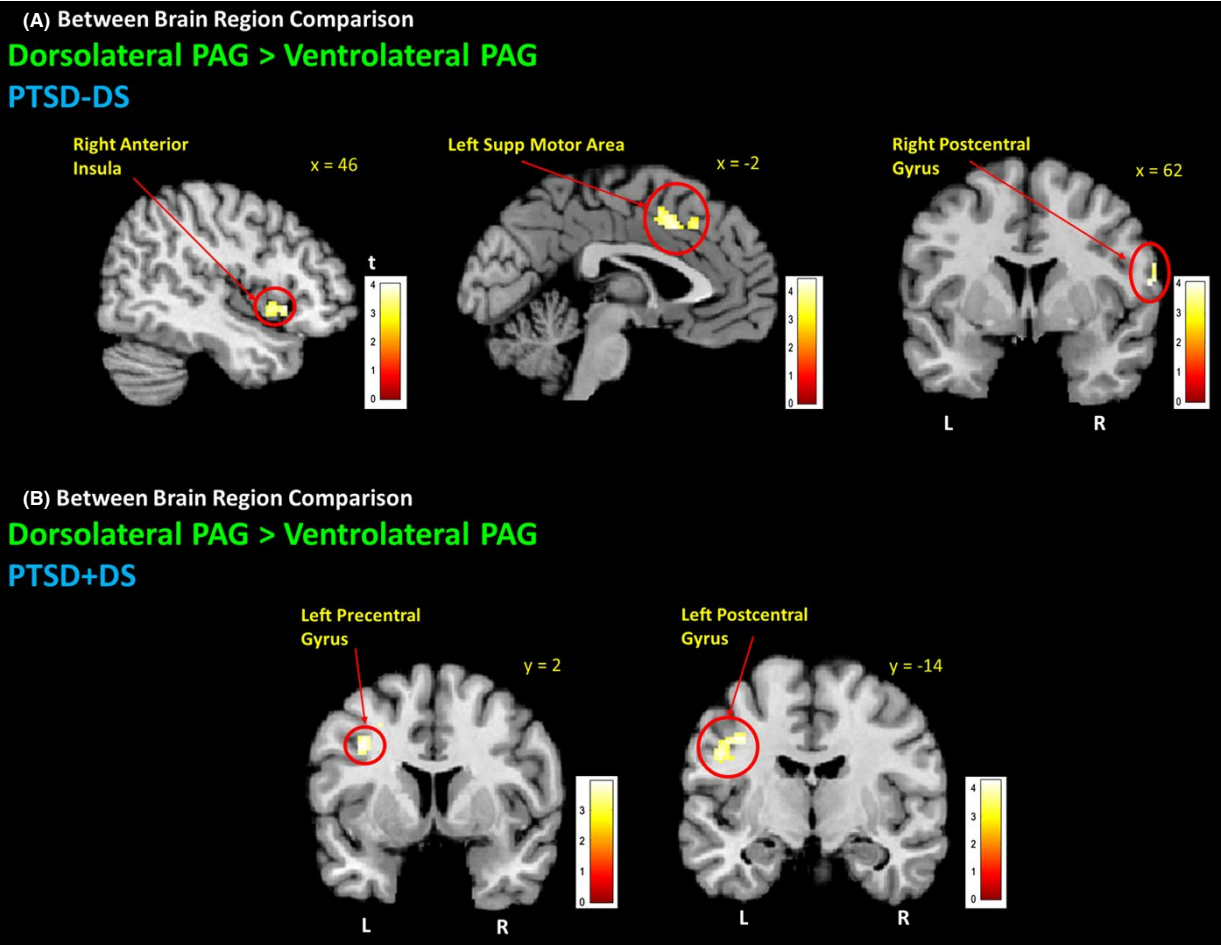
Dorsolateral PAG connectivity with premotor areas during resting state. Both PTSD‐DS and PTSD + DS demonstrated DL‐PAG functional connectivity with premotor areas when comparing DL‐ to VL‐PAG. (A) PTSD‐DS demonstrated greater VL‐PAG connectivity with the right anterior insula, left supplemental motor , and right postcentral gyrus. (B) PTSD + DS demonstrated greater DL‐PAG connectivity with pre‐ and postcentral gyri. FWE whole‐brain cluster corrected at *p *< .05, *k *= 50. PTSD‐DS, nondissociative posttraumatic stress disorder patients; PTSD + DS, dissociative posttraumatic stress disorder patients; DL‐PAG, dorsolateral periaqueductal gray; VL‐PAG, ventrolateral periaqueductal gray; L, left hemisphere; R, right hemisphere

PTSD‐DS subjects showed extensive functional connectivity of both the DL‐PAG and VL‐PAG with the dACC, orbitomedial prefrontal cortex (OMPFC), and bilateral fusiform gyrus (see supplemental results). They also demonstrated DL‐PAG connectivity with cerebellar lobule VI (Fig. 2; Table 2). When comparing DL‐PAG to VL‐PAG connectivity (DL‐PAG > VL‐PAG), PTSD‐DS demonstrated increased functional connectivity with the right anterior insula, the left supplemental motor area, and the right postcentral gyrus (Fig. 3A; Table 3). No greater functional connectivity was observed for the VL‐PAG versus the DL‐PAG (FWE whole‐brain cluster corrected at *p *<* *.05, *k *= 50).

**Table 2:** Post hoc one‐sample *t*‐tests to assess DL‐ and VL‐PAG connectivity patterns within PTSD‐DS and PTSD + DS patient groups (FWE whole‐brain cluster corrected at *p *<* *.05, *k *= 50)

Contrast	L/R	BA	Region	Cluster size	*p* FWE	T voxel	Z‐score	MNI coordinates		
								x	y	z
Within PTSD‐DS DL‐PAG	L		Cerebellar Lobules IV‐V	47,056	<.001	8.30	7.81	−6	−38	−6
	R	54	Hippocampus			7.32	6.99	24	−36	−2
	L	32	Dorsal Anterior Cingulate			6.78	6.51	−10	18	36
	L	37	Fusiform Gyrus			5.78	5.50	−33	−52	−17
	R	37	Fusiform Gyrus	55	.032	5.71	5.54	38	−54	−18
	L	46	Frontal Middle Gyrus	126	<.001	4.74	4.64	−46	48	0
	L	10	Orbitomedial Prefrontal Cortex			4.44	4.35	−40	48	−12
Within PTSD‐DS VL‐PAG	L	20	Inferior Temporal Gyrus	40,354	<.001	7.09	6.79	−48	40	4
	L	48	Caudate			6.82	6.54	−12	6	16
	L	32	Dorsal Anterior Cingulate Cortex			6.39	6.16	−6	26	30
	L		Cerebellar Lobule VI	1187	<.001	5.62	5.46	−22	−54	−28
	L	37	Fusiform Gyrus			5.24	5.11	−44	−48	−20
	R		Cerebellar Vermis			5.10	4.98	2	−54	−6
	L	30	Precuneus	229	<.001	5.46	5.31	−4	−52	14
	L	30	Calcarine Sulcus			5.16	5.03	−8	−58	4
	L	10	Orbitomedial Prefrontal Cortex	168	<.001	5.44	5.30	−42	52	0
	L	19	Lingual Gyrus	77	.006	4.39	4.31	−28	−54	−2
Within PTSD + DS DL‐PAG	L	6	Superior Frontal Gyrus	37,055	<.001	6.40	6.16	−20	−4	50
	L		Cerebellar Lobule VI			6.35	6.13	−32	−54	−22
	R	6	Supplemental Motor			6.32	6.10	12	4	52
	R	8	Dorsal Anterior Cingulate			6.26	6.04	10	12	38
	L	37	Fusiform Gyrus			6.08	5.88	−34	−56	−14
	R	10	Orbitomedial Prefrontal Cortex	182	<.001	4.61	4.52	30	52	−2
Within PTSD + DS VL‐PAG	L	6	Middle Frontal Gyrus	33,686	<.001	6.44	6.20	30	52	−2
	L	37	Fusiform Gyrus			6.28	6.06	−20	−4	48
	L	6	Superior Frontal Gyrus			6.26	6.05	−36	−54	−14
	R	10	Middle Frontal Gyrus	161	<.001	4.41	4.33	−16	4	54
	R	10	Orbitomedial Prefrontal Cortex			4.28	4.20	30	50	−4

PTSD‐DS, nondissociative posttraumatic stress disorder patients; PTSD + DS, dissociative posttraumatic stress disorder patients; DL‐PAG, dorsolateral periaqueductal gray; VL‐PAG, ventrolateral periaqueductal gray; L, left hemisphere; R, right hemisphere; BA, Brodmann Area.

**Table 3:** Post hoc two‐sample *t*‐tests to compare DL‐PAG and VL‐PAG connectivity within PTSD‐DS and PTSD + DS patients (FWE whole‐brain cluster corrected at *p *<* *.05, *k *= 50)

Contrast	L/R	BA	Region	Cluster size	*p* FWE	T voxel	Z‐score	MNI coordinates		
								x	y	z
PTSD‐DS DL > VL‐PAG	L		Cerebellar Lobules IV‐V	414	<.001	7.90	7.48	−6	−38	−6
	R		Cerebellar Vermis			6.02	5.83	6	−42	−8
	L	13	Rolandic Operculum	1131	<.001	5.28	5.15	−40	−28	18
	R	28	Hippocampus	112	.001	5.21	5.08	26	−34	2
	R	44	Inferior Frontal Operculum	141	<.001	4.61	4.51	48	14	2
	R	13	Anterior Insula			3.85	3.79	46	6	−2
	L	28	Hippocampus	72	.009	4.52	4.43	−18	−36	0
	L	32	Dorsal Anterior Cingulate	804	<.001	4.48	4.39	−2	18	38
	L	32	Supplemental Motor			4.43	4.35	−2	10	44
	R	48	Caudate	81	.005	4.41	4.33	16	16	2
	R	49	Putamen			4.21	4.13	26	14	−2
	R	6	Rolandic Operculum	60	.022	4.32	4.24	58	6	10
	R	4	Postcentral Gyrus			4.03	3.97	62	−2	16
PTSD + DS DL > VL‐PAG	R	32	Mid‐Cingulate Gyrus	910	<.001	5.65	5.48	12	−4	44
	R	24	Mid‐Cingulate Gyrus			5.31	5.17	8	−20	46
	L		Cerebellar Lobule IV‐V	546	<.001	5.26	5.13	−8	−46	−10
	L		Cerebellar Vermis			5.26	5.13	−2	−40	−6
	R		Lingual Gyrus	66	.014	4.91	4.80	8	−58	6
	R		Precuneus			4.32	4.24	4	−54	6
	R		Calcarine Sulcus			3.63	3.58	18	−54	6
	L		Frontal Middle Gyrus	238	<.001	4.86	4.76	−28	8	48
	L		Precentral Gyrus			3.93	3.88	−36	2	38
	R		Lingual Gyrus	61	.020	4.66	4.57	26	−52	−10
	R		Parahippocampal Gyrus			3.66	3.61	36	−32	−14
	L		Postcentral Gyrus	181	<.001	4.29	4.21	−38	−14	42
	L		Cerebellar Lobule VI	98	.002	4.24	4.17	−32	−56	−22
	L		Fusiform Gyrus			4.11	4.05	−34	−62	−16
	R		Putamen	66	.014	4.17	4.10	28	−2	6
	L		Putamen	66	.014	4.06	3.99	−24	4	0

PTSD‐DS, nondissociative posttraumatic stress disorder patients; PTSD + DS, dissociative posttraumatic stress disorder patients; DL‐PAG, dorsolateral periaqueductal gray; VL‐PAG, ventrolateral periaqueductal gray; L, left hemisphere; R, right hemisphere; BA, Brodmann Area.

#### 3.3.3 PTSD + DS

PTSD + DS subjects also demonstrated extensive functional connectivity of both the DL‐PAG and VL‐PAG, including the OMPFC, left fusiform gyrus, and cerebellar lobule VI (Fig. 2; Table 2). When comparing DL‐PAG to VL‐PAG connectivity (DL‐PAG > VL‐PAG), PTSD + DS demonstrated increased functional connectivity with the left precentral and postcentral gyri (Fig. 3B; Table 3). No greater functional connectivity was observed for the VL‐PAG versus the DL‐PAG PAG (FWE whole‐brain cluster corrected at *p *<* *.05, *k *= 50).

### 3.4 Functional connectivity differences between control and PTSD patient groups

Between‐group analyses confirmed that there was widespread cortical functional connectivity observed with DL‐PAG and VL‐PAG regions in both PTSD‐DS and PTSD + DS when compared to healthy controls (PTSD‐DS > Control; PTSD + DS > Control; see supplemental results). Moreover, there were no suprathreshold clusters observed where healthy controls exhibited greater functional connectivity than either PTSD patient groups (Control > PTSD‐DS; Control > PTSD + DS) PAG (FWE whole‐brain cluster corrected at *p *< .05, *k *= 50).

**Figure 4:**
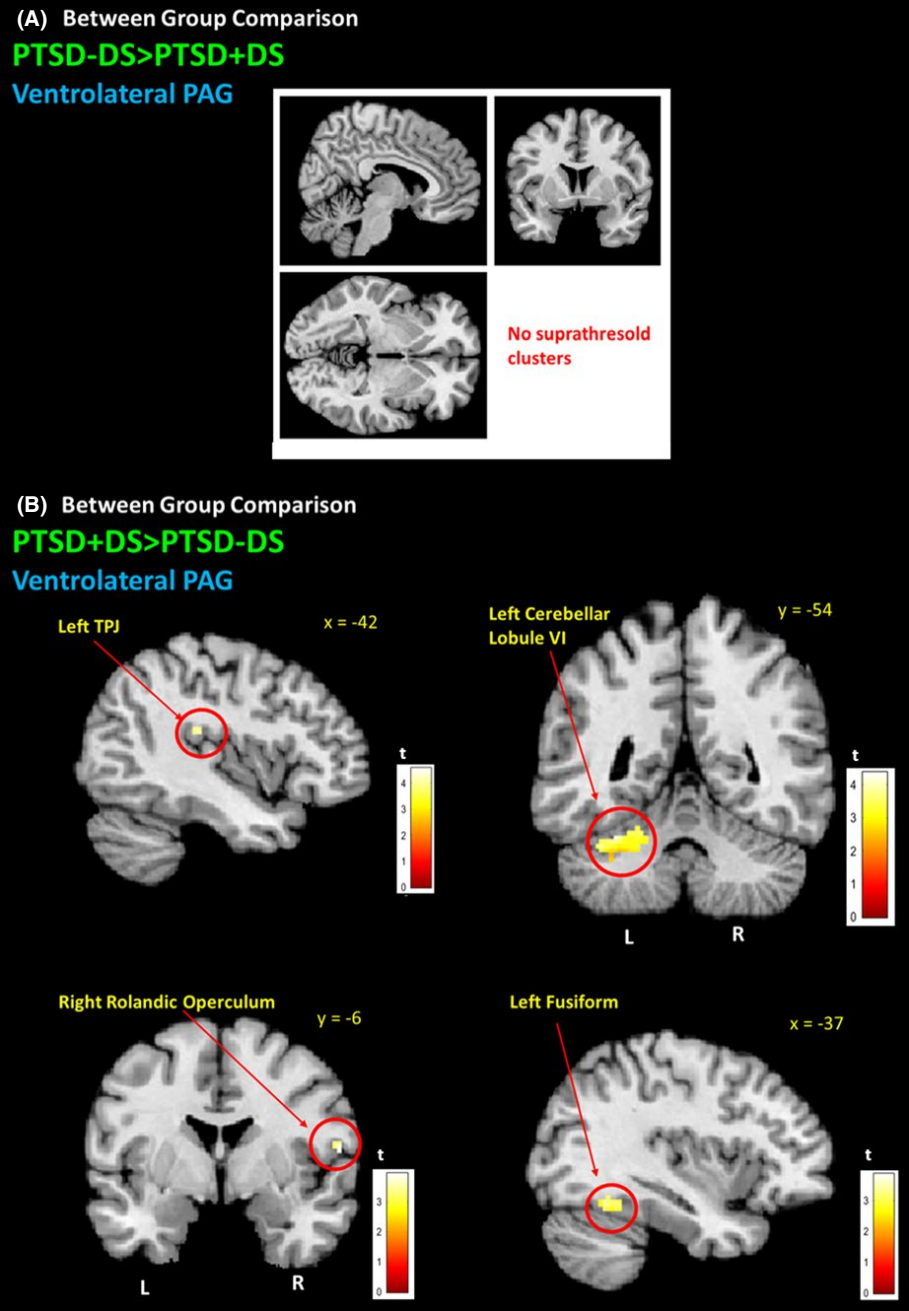
PTSD + DS ventrolateral PAG connectivity with brain areas implicated in depersonalization symptoms. (A) PTSD‐DS did not demonstrate VL‐PAG functional connectivity when compared to PTSD + DS patients. (B) When compared to PTSD‐DS, PTSD + DS demonstrated greater VL‐PAG functional connectivity with the left temporoparietal junction (lTPJ), left cerebellar lobule VI, the right rolandic operculum, and the left fusiformgyrus. FWE whole‐brain cluster corrected at *p *< .05, *k *= 50. PTSD‐DS, nondissociative posttraumatic stress disorder patients; PTSD + DS, dissociative posttraumatic stress disorder patients; DL‐PAG, dorsolateral periaqueductal gray; VL‐PAG, ventrolateral periaqueductal gray; L, left hemisphere; R, right hemisphere

When comparing PTSD‐DS to PTSD+DS (PTSD‐DS>PTSD+DS), there was no greater functional connectivity observed in either PAG subregion (Fig. 4A; Table 4). However, when comparing PTSD + DS to PTSD‐DS (PTSD + DS > PTSD‐DS), greater VL‐PAG connectivity was observed within the right temporoparietal junction, right rolandic operculum, left fusiform gyrus, and cerebellar lobule VI (FWE whole‐brain cluster corrected at *p *<* *.05, *k *= 50) (Fig. 4B; Table 4).

**Table 4:** Post hoc two‐sample *t*‐tests to compare DL‐ and VL‐ PAG connectivity differences between PTSD‐DS and PTSD + DS patients (FWE whole‐brain cluster corrected at *p *<* *.05, *k *= 50)

Contrast	L/R	BA	Region	Cluster size	*p* FWE	T voxel	Z‐score	MNI coordinates		
								x	y	z
PTSD‐DS > PTSD + DS DL‐PAG			No suprathreshold clusters							
PTSD + DS > PTSD‐DS DL‐PAG	L	37	Fusiform Gyrus	148	<.001	4.62	4.52	−36	−56	−12
	L		Cerebellar Lobule VI			4.00	3.94	−32	−54	−22
	L	37	Inferior Temporal Gyrus			3.53	3.49	−48	−50	−14
PTSD‐DS > PTSD + DS VL‐PAG			No suprathreshold clusters							
PTSD + DS > PTSD‐DS VL‐PAG	L	37	Fusiform Gyrus	211	<.001	4.58	4.49	−34	−56	−14
	L		Cerebellar Lobule VI			4.38	4.30	−38	−58	−26
	R	1	Rolandic Operculum	60	.022	3.95	3.89	58	−6	16
	R	44	Precentral Gyrus			3.92	3.64	50	2	22
	R	40	Temporoparietal Junction			3.53	3.49	54	−12	18

PTSD‐DS, nondissociative posttraumatic stress disorder patients; PTSD + DS, dissociative posttraumatic stress disorder patients; DL‐PAG, dorsolateral periaqueductal gray; VL‐PAG, ventrolateral periaqueductal gray; L, left hemisphere; R, right hemisphere; BA, Brodmann Area.

### 3.5 Clinical score correlations with functional connectivity in PTSD patients

CAPS hyperarousal subscale scores were significantly correlated to the functional connectivity between the DL‐PAG and the right fusiform gyrus (*p *=* *.032, FWE cluster corrected; *k* =* *29; *r *= 0.359). Moreover, total CAPS severity score correlated with the functional connectivity between the DL‐PAG and the left fusiform gyrus (*p *=* *.039, FWE cluster corrected; *k* =* *23; *r *= 0.346). No significant correlations were observed between the functional connectivity of the DL‐PAG or VL‐PAG and the CTQ and MDI.

## 4 Discussion

The aim of this study was to compare resting‐state connectivity patterns of the dorsolateral and ventrolateral PAG subdivisions between patients with and without the dissociative subtype of PTSD and controls. In line with our hypotheses, widespread DL‐ and VL‐PAG functional connectivity to brain regions involved in emotional reactivity and defensive action was observed in both PTSD patient groups when compared to healthy participants, suggesting that PTSD patients may exhibit defensive posturing even at rest. Strikingly, even though both PTSD patient groups demonstrated DL‐PAG connectivity to brain regions involved in coordinating active defense ‘fight or flight’ responses (e.g., dorsal anterior cingulate; insula; pre‐/postcentral gyri), only PTSD patients with the dissociative subtype demonstrated greater VL‐PAG connectivity with brain regions related to passive defensive responses and increased levels of depersonalization (left temporoparietal junction, rolandic operculum). We discuss these findings in turn.

### 4.1 PAG connectivity with brain regions involved in autonomic control

Both PTSD‐DS and PTSD + DS groups demonstrated DL‐PAG functional connectivity with the dorsal ACC. In addition, PTSD‐DS patients demonstrated VL‐PAG connectivity with this region. The dACC is an area associated with autonomic control of both the sympathetic nervous system and parasympathetic nervous system, and is implicated in the interpretation of contextual information about the safety of the environment (Bryant et al., 2005; Luu & Posner, 2003; Medford & Critchley, 2010; Shackman et al., 2011). Mobbs et al. (2007) elaborated further on the role of the mid‐dorsal ACC during threat detection, where they demonstrated that increased connectivity between the mid‐dorsal ACC and the midbrain during imminent danger is associated with automatic or ‘hard‐wired’ defensive behaviors (also see Panksepp, 1998). Aberrant ACC activity is strongly associated with PTSD and is thought to contribute to reexperiencing, avoidance, and hyperarousal symptoms (Felmingham et al., 2009; Rougemont‐Bücking et al., 2011; Shin et al., 2001). Given that the PAG failed to show connectivity with the dACC in healthy participants, the increased VL‐PAG and DL‐PAG connectivity with the dACC in PTSD patients observed here suggests inadequate control of fear, which in turn may contribute to a predisposition to engage in reflexive defensive behaviors.

Both PTSD patient groups demonstrated increased DL‐ and VL‐PAG functional connectivity with the orbitomedial prefrontal cortex (see supplemental results) within each patient group and when compared to controls. In support of these findings, Bandler et al. (2000) suggested that the orbitomedial prefrontal cortices are responsible for inputs into autonomic control regions, such as the DL‐ and VL‐PAG, and the hypothalamus.

It is also interesting to note that the areas of interest emerging from the main effect of ROI revealed lateralization to primarily areas in the right hemisphere (see supplemental results), particularly in the right anterior insula, right fusiform gyrus, right temporoparietal junction, and right postcentral gyrus. The medulla oblongata is considered a major autonomic control center in the brainstem (Luiten, ter Horst, Karst, & Steffens, 1985) and is located in the right hemisphere. Accordingly, lateralization to the right brain facilitates ipsilateral connections between autonomic–limbic structures that mediate arousal (i.e., medulla oblongata and right centromedial amydala) (Brake, Sullivan, & Gratton, 2000; Porges, Doussard‐Roosevelt, & Maiti, 1994; Schore, 2002, 2009). These results support the notion that the right brain hemisphere may play a central role in mediating defensive behaviors in both nondissociative and dissociative PTSD patients.

Given our findings of PAG connectivity with the dorsal anterior cingulate and orbitomedial prefrontal cortex in both patient groups, it appears probable that both VL‐PAG and DL‐PAG play a role in autonomic control in both PTSD and its dissociative subtype at rest. This pattern is in contrast to that observed in controls, who did not demonstrate any DL‐ or VL‐PAG connectivity with any of the described areas.

### 4.2 PAG connectivity with the fusiform gyrus

Both PTSD patient groups demonstrated DL and VL‐PAG connectivity with the fusiform gyrus (see supplemental results), supporting Porges’ Polyvagal Theory (2007) that this structure is critical in evaluating faces, movement, and vocalizations to determine whether or not an environment can be perceived as safe or trustworthy (Porges, 2011; also see Adolphs, 2002; Winston, Strange, O'Doherty, & Dolan, 2002). The involvement of the fusiform gyrus in PTSD was supported by our findings, as DL‐PAG connectivity with both the left and right fusiform gyrus correlated with total CAPS and hyperarousal subscale scores, respectively. Given the role of the PAG in detecting threat, these findings may suggest that in contrast to healthy individuals, even during rest, patients with PTSD are consistently evaluating the safety of their environment. Porges’ theorizes further that during threat detection, the fusiform gyrus may initiate top‐down limbic control to generate defensive responses to fear (also see Pessoa, McKenna, Gutierrez, & Ungerleider, 2002). In line with this hypothesis, Williams et al. (2006) reported right amygdala functional connectivity with the fusiform gyrus during conscious attention to fear.

It is interesting to note that this study revealed greater VL‐PAG and DL‐PAG connectivity with the left fusiform gyrus in PTSD + DS as compared to PTSD‐DS. Here, Shaw et al. (2009) found increased activation of the left fusiform gyrus corresponded with inefficient working memory systems in PTSD patients. Indeed, impaired working memory performance has been observed in patients with depersonalization–derealization disorders (Papageorgiou, Lykouras, Ventouras, Uzunoglu, & Christodoulou, 2002). Furthermore, left fusiform gyrus activity appears to vary with high frequency in HRV (Critchley et al., 2003), typically corresponding to increased parasympathetic nervous system activity and bradycardia (Critchley, Corfield, Chandler, Mathias, & Dolan, 2000). Consistent with the defense cascade model (Schauer & Elbert, 2010), these results provide support for the notion that greater VL‐PAG connectivity with the left fusiform gyrus in PTSD + DS may be associated with increased parasympathetic arousal (VL‐PAG connectivity was observed in both PTSD + DS and PTSD‐DS) and could thus contribute to passive defensive strategies through parasympathetic nervous system activity.

PTSD‐DS patients also demonstrated DL‐PAG connectivity with the right fusiform gyrus, a pattern also observed in studies assessing posttraumatic flashbacks often associated with hyperarousal symptoms (Lanius et al., 2006; Osuch et al., 2001). In this study, more severe hyperarousal symptoms were associated with increased DL‐PAG functional connectivity with the right fusiform gyrus. Taken together, these findings suggest that the DL‐PAG may be responsible for initiating hyperarousal responses in PTSD patients that evoke active defensive strategies associated with movement as previously suggested by Bandler et al. (2000), and a corresponding sympathetic ‘fight or flight’ response that occurs despite the absence of an external threat stimulus during resting state.

### 4.3 PAG connectivity with cerebellum

All groups (controls, PTSD + DS, PTSD‐DS+) demonstrated DL‐PAG connectivity with cerebellar lobules IV and V, a structure thought to play a critical role in assessing a trustworthy environment and in fine motor movement (Schutter, 2013; see supplemental results). Critically, this finding is consistent with the Universal Cerebellar Transform theory (Schmahmann, Weilburg, & Sherman, 2007), which suggests the cerebellum may play an unconscious regulatory role in all aspects of brain functioning, including autonomic homeostasis. Although PTSD‐DS and PTSD + DS patient groups demonstrated VL‐PAG functional connectivity with cerebellar lobule VI, connectivity with this structure was greater in PTSD + DS patients. Interestingly, activity in this region was also associated with processing of fearful faces and trauma‐related words in PTSD (Rabellino, Densmore, Frewen, Théberge, & Lanius, 2016). Although autonomic control has been shown to be associated with successful fear conditioning (Critchley, Melmed, Featherstone, Mathias, & Dolan, 2002), cerebellar lobule VI lesions have also been implicated in fear learning during animal studies and thus maintaining unconditioned responses to fear stimuli (i.e., startle response) (Attwell, Cooke, & Yeo, 2002; Bellebaum & Daum, 2011; Lavond & Steinmetz, 1989—see Lange et al., 2015). Our findings suggest that this modulatory role of the cerebellum may be sustained at rest in both controls and PTSD patients. Individuals with PTSD, however, may develop aberrations in PAG‐cerebellar connectivity (i.e., with lobule VI) that may affect the ability of the cerebellum to maintain homeostasis in response to stressors. Future studies are required urgently to confirm this hypothesis.

### 4.4 PAG connectivity with motor regions

In keeping with our hypothesis, both PTSD patient groups demonstrated DL‐PAG connectivity with motor areas thought to be responsible for generating ‘fight or flight’ movements mediated by sympathetic nervous system activity. PTSD‐DS, however, also showed greater DL‐PAG connectivity with the right anterior insula when compared to its VL‐PAG connectivity. This finding points toward a key association between the DL‐PAG and sympathetic nervous system activity, where the right anterior insula is thought to play a critical role in controlling sympathetic arousal in the central autonomic network across numerous studies (Benarroch, 1993; Critchley, 2009; Critchley, Wiens, Rotshtein, Ohman, & Dolan, 2004; de Morree, Rutten, Szabó, Sitskoorn, & Kop, 2016; Saper, 2002). Both PTSD patient groups also demonstrated DL‐PAG connectivity with supplemental premotor areas implicated in generating motor movement.

### 4.5 PAG connectivity with regions involved in depersonalization responses

The PTSD + DS patient group demonstrated greater VL‐PAG connectivity (when compared to PTSD‐DS) with regions associated with depersonalization responses, including the right rolandic operculum and the left temporoparietal junction (TPJ). Interestingly, the rolandic operculum has recently been shown to be a neural correlate of depersonalization in a case of schizotypal disorder (Zaytseva et al., 2015), and is also thought susceptible to alterations stemming from childhood maltreatment (Dannlowski et al., 2012), the prevalence of which is increased in PTSD + DS (Stein et al., 2013). The TPJ is an area implicated in depersonalization experiences, as it is thought to contribute to discrimination between self and nonself (Blanke & Arzy, 2005; Murray, Debbané, Fox, Bzdok, & Eickhoff, 2015). Whereas the right TPJ is important for evaluating self‐location and bodily consciousness (Blanke et al., 2005; Olivé, Tempelmann, Berthoz, & Heinze, 2015), the left TPJ is thought to play an important role in self‐processing, where it may assist in discerning self‐involvement during past autobiographical events (Muscatelli, Addis, & Kensinger, 2010). Here, previous studies examining gray matter alterations as a function of dissociative traits found changes in the inferior parietal cortex, which is also implicated in bodily consciousness (Nardo et al., 2013). Taken together, these findings may help to explain why individuals with PTSD can feel emotionally detached from their traumatic memories during states of depersonalization (Krystal, Bennett, Bremner, Southwick, & Charney, 1995; Lanius et al., 2010; Spiegel, 1997). It should also be noted that these structures contribute to numerous general functions of the brain that are not limited to depersonalization responses; however, the present findings provide a basis for further exploration of PAG functional connectivity in those with dissociative traits in order to further delineate neural correlates associated with depersonalization responses.

### 4.6 Limitations and conclusions

Some limitations of this study need to be considered. Firstly, we did not include a trauma‐exposed control group without PTSD, as individuals with matching trauma histories often meet lifetime criteria for one or more psychiatric disorders. Although previous studies have also reported gender‐related differences during resting state in healthy individuals (Gur et al., 1995; Tian, Wang, Yan, & He, 2011), these results are conflicting, with numerous authors suggesting it is not necessary to control for gender (Damoiseaux et al., 2006; Weissman‐Fogel, Moayedi, Taylor, Pope, & Davis, 2010). Unfortunately, this study was not powered to examine gender differences, which have been observed in a previous pain‐related functional PAG connectivity study (Linnman, Beucke, Jensen, Gollub, & Kong, 2011); this issue warrants exploration in future studies as these differences may influence clinical symptoms. Furthermore, additional measures designed to reduce physiological noise from the fMRI scanner, such as a component‐based approach, should be examined in future studies (Behzadi, Restom, Liau, & Liu, 2007). It is also important to note that this study is cross‐sectional in nature and can therefore not make conclusions about cause and effect. Finally, future studies should also explore the use of a greater magnetic field strength to explore PAG subdivision functional connectivity with greater temporal resolution and assess how these patterns of functional connectivity may vary in response to a stressor, as demonstrated in previous animal studies (Adamec, Berton, & Abdul‐Razek, 2009).

On balance, this study reveals novel findings highlighting the importance of examining altered subcortical functional connectivity networks in PTSD patients and its dissociative subtype during resting state. Even during resting state, patients with PTSD showed extensive VL‐PAG and DL‐PAG functional connectivity with areas associated with emotional reactivity and defensive action. It is possible that these findings reflect greater defensive posturing observed in PTSD even at rest. Our findings further indicate that patients with the dissociative subtype of PTSD show unique patterns of PAG functional connectivity when compared to those without the subtype. Here, although all patients with PTSD demonstrated DL‐PAG functional connectivity with areas linked to hyperarousal and the initiation of active coping strategies through sympathetic nervous system activation (e.g., dACC; right insula; pre‐/postcentral gyri), only PTSD patients with the subtype demonstrated greater VL‐PAG functional connectivity with brain regions associated with passive coping strategies and increased levels of depersonalization (e.g., left TPJ; right rolandic operculum; left fusiform gyrus). Taken together, these findings represent an important first step to identifying neural and behavioral targets for therapeutic interventions that address both active and passive defensive strategies in trauma‐related disorders.

## Conflicts of Interest

None declared.
