# Supplementary material for: fMRI functional connectivity of the periaqueductal gray in PTSD and its dissociative subtype
Source: Brain Behav. 2016 Sep 20;6(12):e00579. doi: 10.1002/brb3.579 (PMC5167004; doi:10.1002/brb3.579)
Supplement: Supplementary file 1 [file BRB3-6-e00579-s001.pdf]

Supplemental Results

Supplemental Table 1 (see captions below)

Contrast	L/R	BA	Region	Cluster Size	p FWE	Z-Score	MNI Coordinates x	y	z
Group x ROI Interaction	R	6	Dorsal Anterior Cingulate Cortex	231	<0.001	4.81	12	-6	44
	R	6	Supplemental Motor			4.34	4	-10	54
	R	31	Mid-Cingulate Cortex			4.13	6	-20	46
	L	6	Pre-central Gyrus	62	0.004	4.16	-38	0	36
Main Effect of Group	L	6	Frontal Middle Gyrus	45514	<0.001	7.24	-26	-6	52
	L	24	Mid-Cingulate Gyrus			6.32	-4	16	36
	L	10	Frontal Middle Gyrus	50	0.014	4.27	-40	56	2
	L	10	Orbitomedial Prefrontal Cortex			4.06	-44	50	-4
Main Effect of PAG Subdivision	L		Cerebellar Lobules IV-V	915	<0.001	65535	-6	-38	-6
	R	23	Calcarine Sulcus	55	0.016	5.60	4	-56	12
	R	30	Calcarine Sulcus			3.69	16	-54	6
	R	24	Dorsal Anterior Cingulate	1703	<0.001	5.26	10	-20	44
	L	6	Supplemental Motor Area			5.09	-4	8	48
	L	41	Heschl Gyrus			5.08	-38	-26	14
	R	28	Hippocampus	457	<0.001	5.26	20	-34	0
	R	35	Parahippocampal Gyrus			4.78	36	-30	-14
	R	13	Anterior Insula	159	<0.001	4.85	48	2	0
	R	22	Superior Temporal Pole			4.10	56	10	-6
	R	8	Frontal Middle Gyrus	156	<0.001	4.52	32	14	36
	R	9	Frontal Middle Gyrus			4.11	30	30	34
	R	44	Inferior Frontal Operculum			4.09	42	12	34
	R	32	Dorsal Anterior Cingulate	77	0.002	4.24	12	24	36
	R	8	Superior Frontal Gyrus			3.69	6	28	44
	R	22	Superior Temporal Gyrus	58	0.012	4.21	50	-28	-2
	L	44	Anterior Insula	65	0.006	4.08	-36	14	12
	R	44	Middle Insula	69	0.005	4.08	34	6	14

Supplemental Figure 1

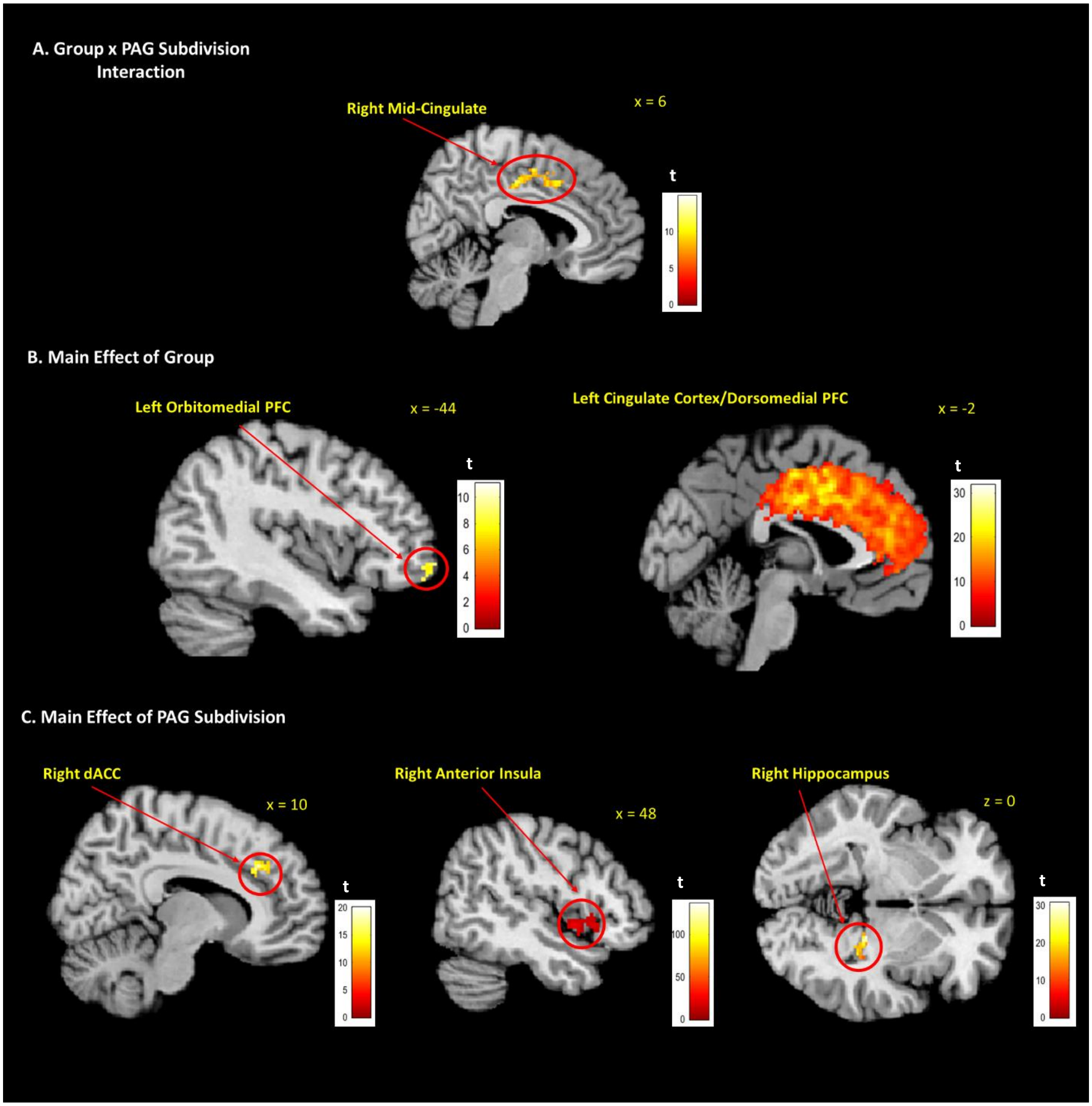

Supplementary Table 2

Contrast	L/R	BA	Region	Cluster Size	p FWE	Z-Score	MNI Coordinates x	y	z
**Within Control Group – DL-PAG**	L		Cerebellar Lobules IV-V	135	<0.001	Inf	-6	-38	-6
	R		Cerebellar Lobule III			4.66	8	-40	-8
	L		Cerebellar Vermis			4.42	0	-46	-6
**Within Control Group – VL-PAG**			No suprathreshold clusters						
**Control DL>VL-PAG**	L		Cerebellar Lobules IV-V	61	0.020	6.89	-6	-38	-6
	L		Cerebellar Vermis			3.89	0	-50	-10
**Control DL>VL-PAG**			No suprathreshold clusters						
**Control>PTSD-DS DL-PAG**			No suprathreshold clusters						
**Control>PTSD+DS DL-PAG**			No suprathreshold clusters						
**Control>PTSD-DS VL-PAG**			No suprathreshold clusters						
**Control>PTSD+DS VL-PAG**			No suprathreshold clusters						

Supplementary Table 3

Contrast	L/R	BA	Region	Cluster Size	p FWE	Z-Score	MNI Coordinates x	y	z
PTSD-DS>Control DL-PAG	L	44	Pre-central Gyrus	26318	<0.001	6.43	-58	8	24
	R	13	Rolandic Operculum			6.08	40	-8	18
	L	24	Dorsal Anterior Cingulate Cortex			5.88	0	-10	38
	R	54	Hippocampus	234	<0.001	6.04	24	-36	-2
	R		Thalamic Pulvinar			4.00	20	-32	12
	R		Cerebellar Lobules IV-V	595	<0.001	5.41	14	-56	-14
	R		Cerebellar Vermis			4.75	4	-52	-6
	L		Cerebellar Lobules IV-V			4.74	-12	-56	-14
	R	18	Calcarine Sulcus	102	<0.001	4.61	22	-60	4
	R	23	Calcarine Sulcus			4.15	12	-56	12
	R	30	Lingual Gyrus			3.84	8	-62	4
	R	40	Rolandic Operculum	70	0.011	4.54	48	-20	16
	R	41	Heschl Gyrus			3.75	52	-16	10
	R	41	Superior Temporal Gyrus			3.40	50	-30	12
	L	21	Middle Temporal Gyrus	55	0.032	4.26	-50	2	-30
	L	38	Middle Temporal Pole			3.46	-40	8	-30
PTSD-DS>Control VL-PAG	R	6	Mid-Cingulate Gyrus	18666	<0.001	6.24	-20	0	42
	R	23	Mid-Cingulate Gyrus			5.67	2	-18	34
	R	6	Supplemental Motor			5.64	12	0	56
	R		Cerebellar Lobule VI	55	0.032	5.57	18	-64	-18
	L	18	Lingual Gyrus	157	<0.001	5.18	-16	-62	2
	L	23	Precuneus			4.36	-4	-54	18
	L	23	Calcarine Sulcus			3.69	-12	-58	10
	R	54	Hippocampus	134	<0.001	4.71	32	-36	-8
	R	18	Lingual Gyrus			4.04	22	-48	0
	L	43	Rolandic Operculum	55	0.032	4.67	-44	-14	22
	L		Cerebellar Vermis	104	0.001	4.47	-2	-58	-10
	L		Cerebellar Lobules IV-V			4.30	-6	-58	-18
	L	10	Orbitomedial Prefrontal Cortex	84	0.004	4.06	-40	48	-10

Supplementary Table 4

Contrast	L/R	BA	Region	Cluster Size	p FWE	Z-Score	MNI Coordinates x	y	z
**PTSD+DS>Control DL-PAG**	L	6	Pre-central Gyrus	26486	<0.001	6.09	-28	-12	48
	L	24	Frontal Middle Gyrus			5.94	-30	8	42
	R	6	Supplemental Motor			5.85	10	4	52
	R	10	Frontal Middle	110	0.001	4.06	32	58	-2
	R	11	Orbitomedial Prefrontal			6.58	26	48	-12
**PTSD+DS>Control VL-PAG**	L	6	Frontal Middle Gyrus	23618	<0.001	5.99	-26	-10	48
	R	6	Dorsal Anterior Cingulate Cortex			5.35	14	28	42
	R	21	Middle Temporal Gyrus	770	<0.001	4.92	44	-40	-2
	R	19	Lingual Gyrus			4.83	20	-54	-8
	R	38	Superior Temporal Pole	110	0.001	4.43	52	16	-16
	R	10	Superior Orbitofrontal Gyrus	112	0.001	3.96	30	56	-4
	R	10	Orbitmedial Prefrontal Cortex			3.64	22	58	-12

Supplemental Figure 2

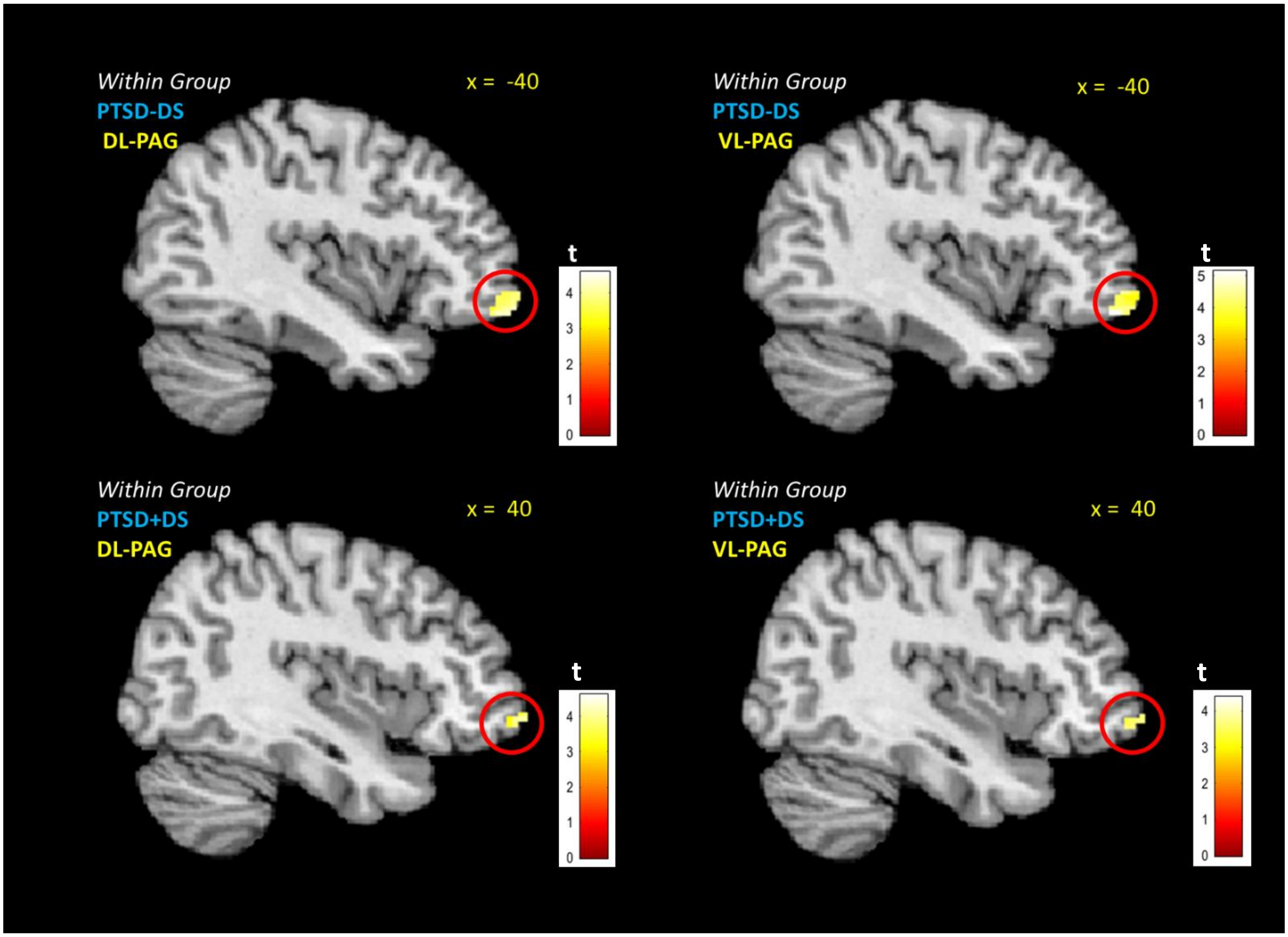

Supplemental Figure 3

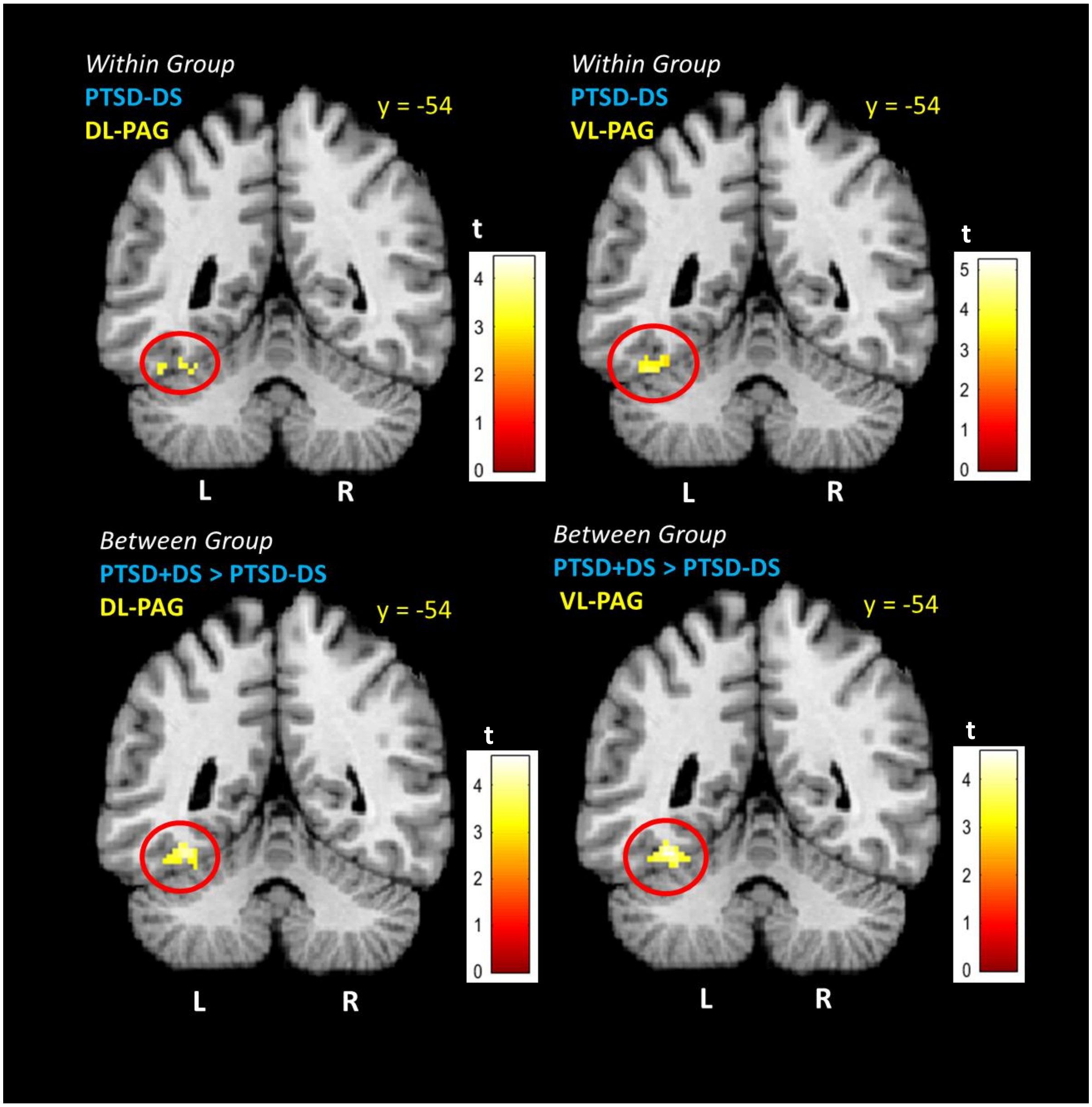

Supplemental Figure 4

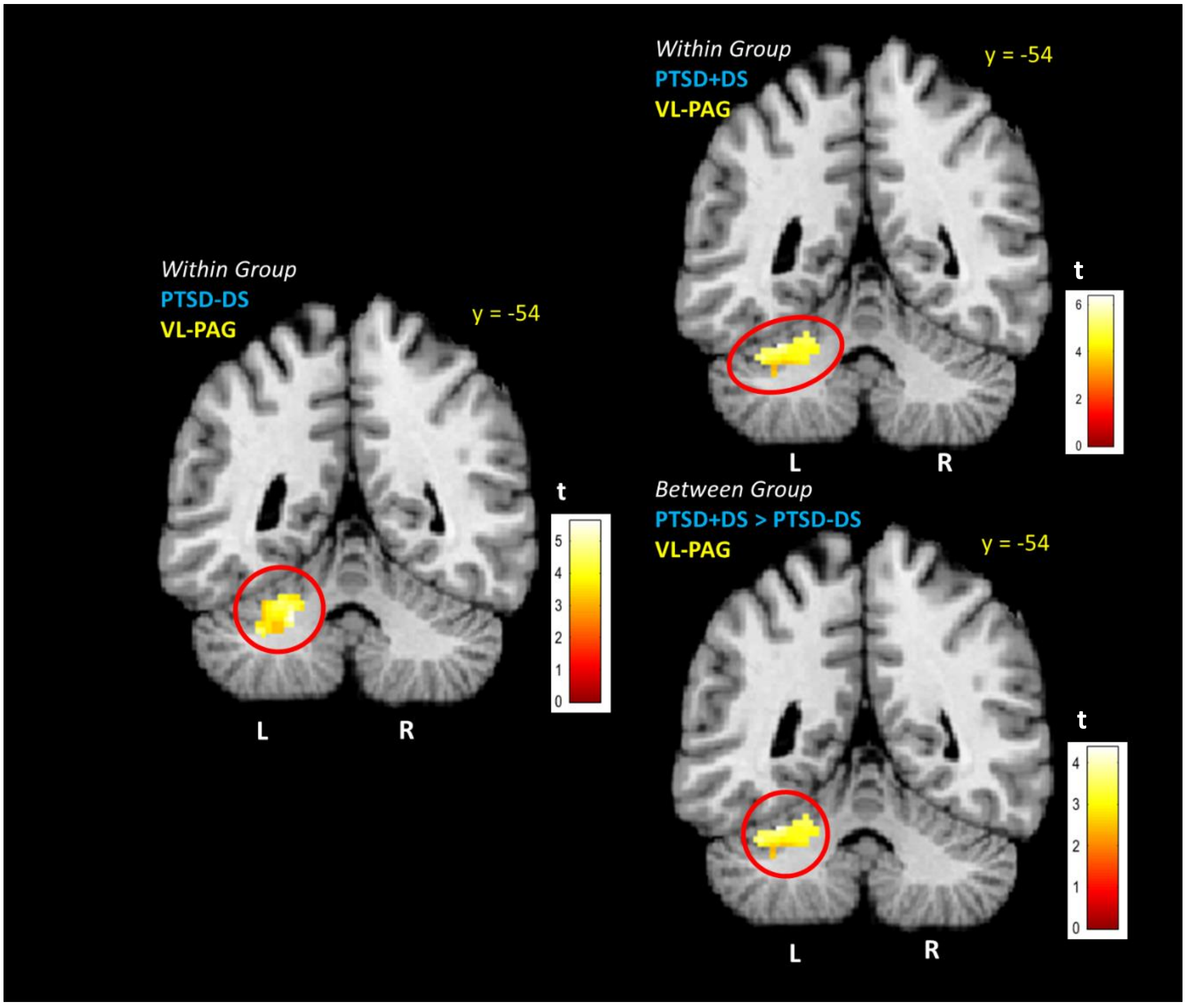

Supplemental Table 1. 3 (Group) x 2 (PAG subdivision) Full Factorial ANOVA. Areas revealed in the full-factorial interaction between group (Control, PTSD-DS, PTSD+DS) and PAG subdivision (DL- and VL-PAG), as well as the main effects for each factor. Abbreviations: L/R, left or right hemispheres; BA, Brodmann area. Full factorial analysis of variance displayed FWE whole brain corrected clusters at $p<0.05$, k=50.

Supplemental Figure 1. Brain areas revealed in the full-factorial analysis. (*A*) The mid-cingulate gyrus was revealed in the interaction between participant group (control, PTSD-DS, PTSD+DS) and PAG subdivision (DL- and VL-PAG). (*B*) Brain areas for the main effect of group include the left orbitomedial prefrontal cortex (PFC) and widespread cingulate cortex/dorsolateral PFC connectivity (dorsal/ventral ACC, midcingulate). (C) The brain areas observed for the PAG subdivision main effect were concentrated largely in the right hemisphere, including the dACC, anterior insula and hippocampus. Anatomical labels were determined using AAL atlas. FWE whole brain cluster corrected at $p<0.05$, k=50. Abbreviations: PTSD-DS, non-dissociative posttraumatic stress disorder patients; PTSD+DS, dissociative posttraumatic stress disorder patients; DL-PAG, dorsolateral periaqueductal gray; VL-PAG, ventrolateral periaqueductal gray.

Supplementary Table 2. Post-hoc one-sample and two-sample t-tests of within- and between-group comparisons of healthy control participants to PTSD-DS and PTSD+DS patient groups. Full factorial analysis of variance displayed FWE whole brain corrected clusters at $p<0.05$, k=50. Abbreviations: PTSD-DS, non-dissociative posttraumatic stress disorder patients; PTSD+DS, dissociative posttraumatic stress disorder patients; DL-PAG, dorsolateral periaqueductal gray; VL-PAG, ventrolateral periaqueductal gray; L/R, left or right hemispheres; BA, Brodmann area.

Supplementary Table 3. Post-hoc two-sample t-tests that assess between-group comparisons of PTSD-DS to healthy controls.

Abbreviations: PTSD-DS, non-dissociative posttraumatic stress disorder patients; DL-PAG, dorsolateral periaqueductal gray; VL-PAG, ventrolateral periaqueductal gray; L/R, left or right hemispheres; BA, Brodmann area. FWE whole brain cluster corrected at $p<0.05$, k=50.

Supplementary Table 4. Post-hoc two-sample t-tests that assess between-group comparisons of PTSD+DS to healthy controls.

Abbreviations: PTSD+DS, dissociative posttraumatic stress disorder patients; L/R, left or right hemispheres; BA, Brodmann area. FWE whole brain cluster corrected at $p<0.05$, k=50.

Supplemental Figure 2. Orbitomedial Prefrontal Cortex. PTSD-DS and PTSD+DS patient groups demonstrated both DL- and VL-PAG functional connectivity with the orbitomedial prefrontal cortex (red circles) during resting state. FWE whole brain cluster corrected at $p<0.05$, k=50.

Abbreviations: PTSD-DS, non-dissociative posttraumatic stress disorder patients; PTSD+DS, dissociative posttraumatic stress disorder patients; DL-PAG, dorsolateral periaqueductal gray; VL-PAG, ventrolateral periaqueductal gray.

Supplemental Figure 3. Fusiform Gyrus. Both PTSD-DS and PTSD+DS demonstrated both DL- and VL-PAG functional connectivity with the left fusiform gyrus (red circles) during resting state. PTSD-DS also demonstrated connectivity with the right fusiform gyrus (not shown). FWE whole brain cluster corrected at $p<0.05$, k=50.

Abbreviations: PTSD-DS, non-dissociative posttraumatic stress disorder patients; PTSD+DS, dissociative posttraumatic stress disorder patients; DL-PAG, dorsolateral periaqueductal gray; VL-PAG, ventrolateral periaqueductal gray; L, left hemisphere; R, right hemisphere.

Supplemental Figure 4. Cerebellar Lobule VI. PTSD-DS demonstrated VL-PAG functional connectivity with cerebellar lobule VI (red circles), however PTSD+DS demonstrated both DL- and VL-PAG functional connectivity in the same area. PTSD+DS showed greater vlPAG functional connectivity with lobule VI when compared to PTSD-DS. Full factorial analysis of variance displayed FWE whole brain corrected clusters at $p<0.05$, k=50.

Abbreviations: PTSD-DS, non-dissociative posttraumatic stress disorder patients; PTSD+DS, dissociative posttraumatic stress disorder patients; DL-PAG, dorsolateral periaqueductal gray; VL-PAG, ventrolateral periaqueductal gray; L, left hemisphere; R, right hemisphere.
